# Filling the data gaps on sickle cell anaemia in sub-Saharan Africa

**DOI:** 10.1016/S2352-3026(22)00042-4

**Published:** 2022-03-01

**Authors:** Thomas Neil Williams

**Affiliations:** Institute of Global Health Innovation, Department of Surgery and Cancer, Imperial College London, London SW7 2BX, UK

**Figure:**
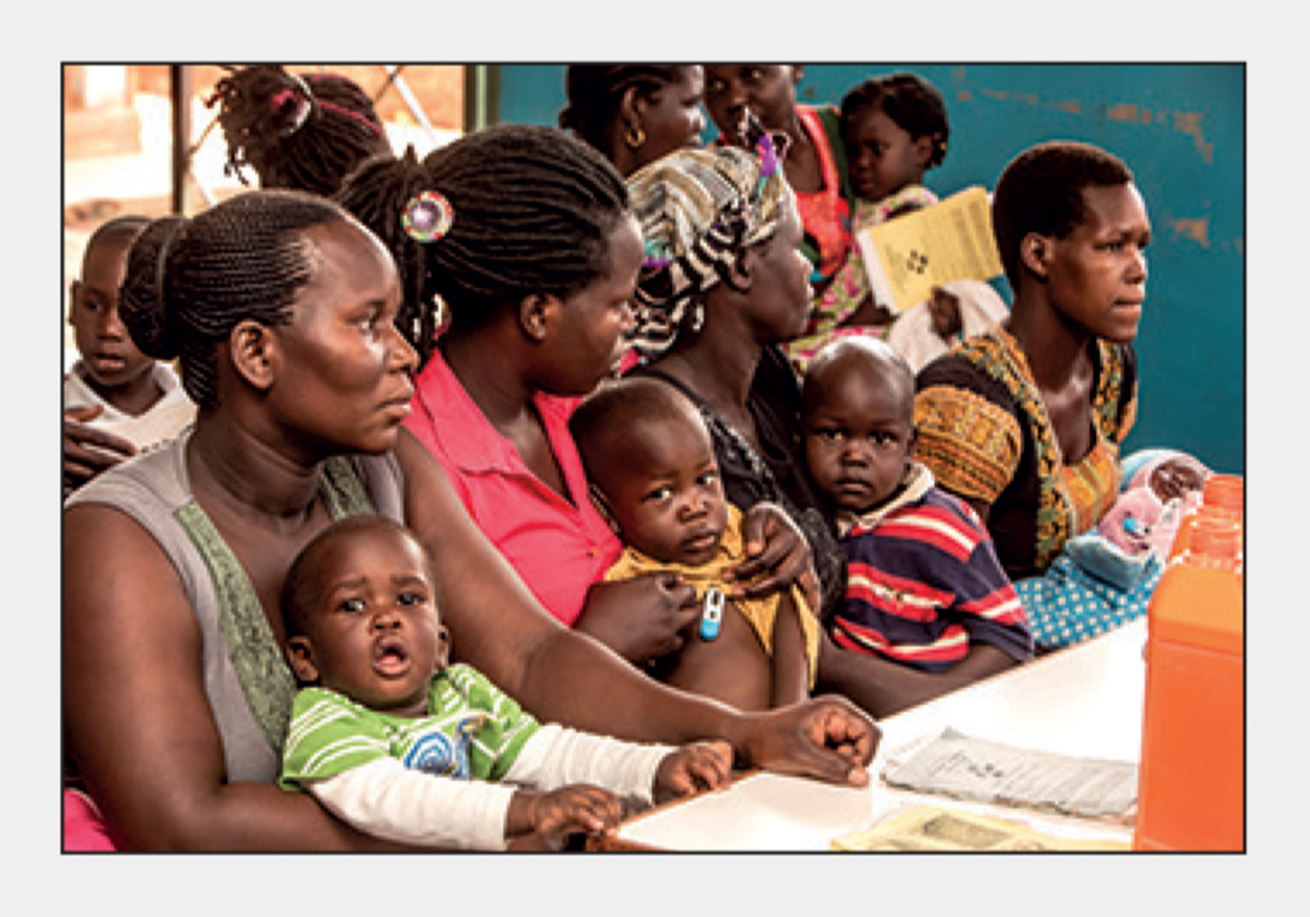


Although sub-Saharan Africa is the epicentre of sickle cell anaemia globally, until recently, the disease has been widely neglected within the region. During the late 1990s, mortality in children younger than 5 years in sub-Saharan Africa was close to 200 per 1000 livebirths,^1^ and consequently, an incurable disease such as sickle cell anaemia affecting ten per 1000 children was not a priority. However, with many sub-Saharan African countries going through a period of epidemiological and demographic transition, overall child mortality is declining rapidly and provision of better care for children born with sickle cell anaemia has now become a necessity.^2,3^

An early priority when advocating for any disease are high quality data. However, reliable data on sickle cell anaemia in sub-Saharan Africa are scarce. The absence of newborn screening, national registries, and computerised hospital record systems mean that few reliable data are available regarding even the most basic questions, including the number of babies born with sickle cell anaemia and their rates of survival. For example, the best estimate of the total number of children born with sickle cell anaemia globally used indirect methods based on mathematical models that incorporate historical data on carrier frequencies and contemporary data on regionally specific population densities and total birth rates.^4^ Similarly, the most widely cited study^5^ on death rates in children with sickle cell anaemia, which suggested a mortality rate of 50−90% for children younger than 5 years during the past half century, was based on a review of published surveys that included data on the prevalence of sickle cell disease stratified by age. Although this study was valuable, more accurate methods are needed to inform current health policy in sub-Saharan Africa at the national and subnational level. One approach is to use birth cohorts, but these are expensive to set up and require long follow-up to yield informative data.

In *The Lancet Haematology*, Brigitte Ranque and colleagues^6^ describe a novel approach to estimate mortality among children born with sickle cell anaemia. The authors’ method holds promise for deriving locally applicable data more cheaply and quickly than the use of direct methods. Sickle cell anaemia is an autosomal recessive condition that results from two heterozygous parents. On the basis of Mendel’s law, each child born to such parents has a 25% chance of having sickle cell anaemia, a 25% chance of being genetically healthy, and a 50% chance of being a carrier. The authors applied this law in a retrospective, multicentre study and recruited mothers of children with confirmed sickle cell anaemia who were attending clinics in five countries in sub-Saharan Africa. Mothers who had at least four children, including the index child, born more than 5 years ago, were interviewed to establish how many of their non-index children had died. Importantly, mothers were also asked to confirm that all children were conceived from the same father. The resulting rates were then inflated to account for the statistical chance that each child (alive or deceased) had sickle cell disease, following Mendel’s law. To compare these rates with children who did not have sickle cell anaemia, the authors collected similar data from control mothers who were neighbours or relatives of mothers already in the study (as part of families with cases of sickle cell anaemia) and were neither heterozygous or homozygous for sickle cell anaemia (AA phenotype), as confirmed by point-of-care testing. The results suggest that rates of mortality in children younger than 5 years in this study (36·4%, 95% CI 33·4−39·4)^6^ are broadly similar to those in a recently published cohort study,^7^ and are 5·35 (4·68−6·12) times higher than those seen in children without sickle cell anaemia.

This new method offers potential advantages over the observation of cohorts recruited at birth, most notably the high costs and long observation period that are needed for cohort observation. However, the logistical and ethical challenges of the new approach are not entirely trivial. The study by Ranque and colleagues^6^ was conducted in 1315 families with cases of sickle cell anaemia and 1243 control families, each confronted by emotionally challenging issues. Moreover, the stigma associated with sickle cell anaemia meant that many mothers were reluctant to give contact details of potential neighbours who could be recruited to the control group, which was a cause for possible bias. Other potential biases, not discussed by the authors in particular detail, include the fact that 50% of children born to heterozygous parents will also be carriers. Such children are healthier than children who do not carry the sickle cell gene because of the protection from direct and indirect consequences of malaria caused by *Plasmodium falciparum.*^8^ As a result, the mortality rates presented for children with sickle cell anaemia could be greatly underestimated. Moreover, the reliance on maternal history regarding non-paternity might not be entirely reliable, and is also a potential cause for substantial bias. Despite these caveats, even at a country-specific level, the study produced plausible estimates with reasonable CIs from surveys, involving as few as 190 families with cases of sickle cell anaemia in Côte d’Ivoire, one of the countries involved. Therefore, this new approach offers an attractive short-term solution to the documentation of mortality rates of children with sickle cell anaemia that could form a baseline from which to judge the impact of specific future interventions.
